# Exploring Embodied Intelligence in Soft Robotics: A Review

**DOI:** 10.3390/biomimetics9040248

**Published:** 2024-04-19

**Authors:** Zikai Zhao, Qiuxuan Wu, Jian Wang, Botao Zhang, Chaoliang Zhong, Anton A. Zhilenkov

**Affiliations:** 1 HDU-ITMO Joint Institute, Hangzhou Dianzi University, Hangzhou 310018, China; zhaozikai2022@163.com (Z.Z.);; 2 Institute of Electrical Engineering, School of Automation, Hangzhou Dianzi University, Hangzhou 310018, China; 3 International Joint Research Laboratory for Autonomous Robotic Systems, Hangzhou 310018, China; 4 Institute of Hydrodynamics and Control Processes, Saint-Petersburg State Marine Technical University, 190121 Sankt-Petersburg, Russia; zhilenkovanton@gmail.com

**Keywords:** embodied intelligence, bio-inspired, soft robotics, reinforcement learning

## Abstract

Soft robotics is closely related to embodied intelligence in the joint exploration of the means to achieve more natural and effective robotic behaviors via physical forms and intelligent interactions. Embodied intelligence emphasizes that intelligence is affected by the synergy of the brain, body, and environment, focusing on the interaction between agents and the environment. Under this framework, the design and control strategies of soft robotics depend on their physical forms and material properties, as well as algorithms and data processing, which enable them to interact with the environment in a natural and adaptable manner. At present, embodied intelligence has comprehensively integrated related research results on the evolution, learning, perception, decision making in the field of intelligent algorithms, as well as on the behaviors and controls in the field of robotics. From this perspective, the relevant branches of the embodied intelligence in the context of soft robotics were studied, covering the computation of embodied morphology; the evolution of embodied AI; and the perception, control, and decision making of soft robotics. Moreover, on this basis, important research progress was summarized, and related scientific problems were discussed. This study can provide a reference for the research of embodied intelligence in the context of soft robotics.

## 1. Introduction

Over the recent 50 years, artificial intelligence has undergone tremendous changes, transforming from a computational discipline into a highly interdisciplinary field encompassing many areas. In 1950, Alan Mathison Turing first proposed the concept of embodied intelligence [1]. Since then, developing effective methods for creating systems with embodied intelligence has been one of the primary goals of artificial intelligence. In the 1960s, researchers began to explore the possibility of artificial intelligence embodiment. Philosopher and neuroscientist Francisco Varela introduced the philosophy of the embodied mind, suggesting that human cognitive intelligence should be understood from the perspective of the human body and its interaction with the physical environment. In the 1970s, the Stanford Research Institute in the United States developed the first mobile robot, Shakey, capable of autonomously perceiving, modeling the environment, planning behavior, and executing tasks. The most influential figure in developing embodied intelligence as a method for designing intelligent robots is Rodney Brooks, who proposed designing intelligent machines through perceptual and action-driven environmental interaction rather than through predefined instruction codes [2]. Based on this theory, artificial intelligence can be divided into non-embodied and embodied intelligence. Non-embodied intelligence, also known as disembodied intelligence, typically refers to software programs, algorithms, and other digital entities that operate within computers and do not need to interact with the external world through physical components. This type of non-embodied intelligence has achieved great success, which was driven by the development of internet information processing, big data, and GPU resources [3].

Embodied intelligence refers to artificial intelligence systems that possess a physical presence, like robots, which can perceive the environment through sensors and directly interact with the environment through physical means, such as actuators, thereby making decisions and taking actions. Of course, embodied intelligence and non-embodied intelligence are not mutually exclusive, especially regarding methods. Current techniques such as machine learning, reinforcement learning, and transfer learning can all serve as tools for studying embodied intelligence. Embodied intelligence is one of the critical lessons we can learn from nature to improve the behavior of robots [4]. We observe in nature the smooth, fast, and efficient actions of organisms in complex environments. In the field of soft robotics, we are mimicking these organisms in nature to provide solutions for achieving embodied intelligent robots. In the recent decade, soft robotics has experienced rapid development, including advances in new control mechanisms [5], progress in kinematics and dynamics modeling, and the utilization of innovative materials and designs (such as soft silicone, hydrogels, shape memory alloys, liquid metals), enabling the production of flexible and adaptable components with excellent performance. Soft robotics has broad application prospects in reconnaissance, detection, rescue, and medical fields. These areas typically require robots with adaptive capabilities to interact with constantly changing environments, make autonomous decisions, and change shape. Their capability to solve unconventional tasks is closely related to the goals of embodied intelligence research. The intersection and fusion of embodied intelligence and soft robotics enable researchers to explore more innovation and application areas.

The principles dictated by nature offer us lessons that we can learn. In the current context of soft robotics, embodied intelligence encompasses the comprehensive integration of principles from the field of biomimicry regarding morphology, structure, and control; evolutionary insights from the field of computational intelligence; and relevant research outcomes from machine learning concerning perception, learning, and decision making. This amalgamation has formed a relatively complete, independent, and rapidly evolving discipline branch. Although there have been some publications with “embodied intelligence” as their central theme, their content mainly focused on the integration of perception and behavior while neglecting the influence of physical morphology [6,7,8]. In embodied intelligence, the relationship between the physical morphology of the agent and perception, learning, and control plays a crucial role [9]. Embodied intelligence within the context of soft robotics is a topic worthy of discussion. This article comprehensively analyzes and summarizes significant research advancements related to this issue, aiming to provide references for the development of this field.

This article is structured as follows: Section 2 elaborates on the research methods. Section 3 discusses the relationship between embodied intelligence and other intelligent systems. Section 4 provides an overall introduction to the latest research advancements in embodied intelligence within the field of soft robotics. Section 4.1, Section 4.2 and Section 4.3 provide detailed reviews of advanced research in embodied morphological computation, embodied artificial evolution, and the perception, control, and decision making in soft robots. Section 5 revisits the core insights of this literature review and delves into the potential trends and opportunities for future research on embodied intelligence.

## 2. Methodology

### 2.1. Search Strategy

**Figure 1:**
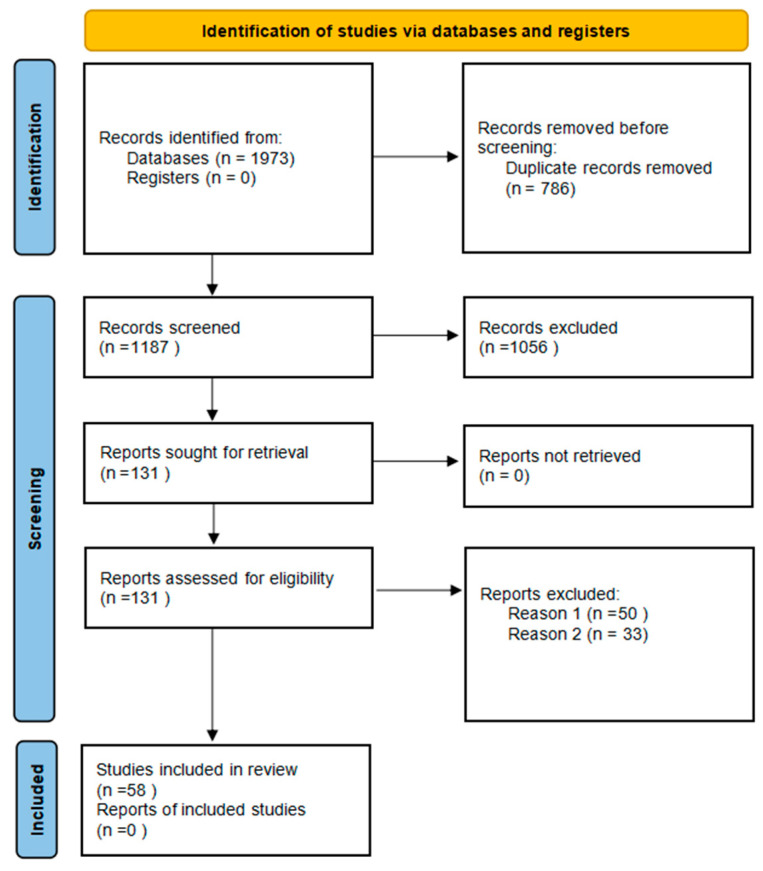
PRISMA diagram of literature search [10].

We performed a systematic search of the literature using online databases, including Web of Science, Scopus, and IEEE Xplore, from inception to March 2024. The search included the following keywords and phrases: (soft robot OR bionic robot) AND (embodied intelligence OR physical intelligence OR computational intelligence OR perceptual intelligence OR morphological intelligence) AND (biological evolution OR environment interactions OR autonomous decision-making OR control strategy). These keywords were searched within the titles, abstracts, and keywords of the papers. The article selection process, following PRISMA guidelines for systematic review updates, is depicted in Figure 1.

### 2.2. Pre-Inclusion and Exclusion Criteria

We applied the following pre-inclusion and exclusion criteria:

1. The paper does not clearly discuss the application of artificial intelligence technologies in soft robotics or how artificial intelligence extends to embodied intelligence.
2. The research focus deviates from soft robotics or embodied intelligence, such as concentrating solely on rigid robots, or the study does not cover enhancing robot intelligence through an interaction with the environment.

### 2.3. Screening and Selection Process

After the initial search, the papers were screened based on their titles and abstracts to ensure they met the pre-inclusion criteria. The full text of the selected papers was then assessed for eligibility based on the exclusion criteria.

### 2.4. Data Extraction and Analysis

**Figure 2:**
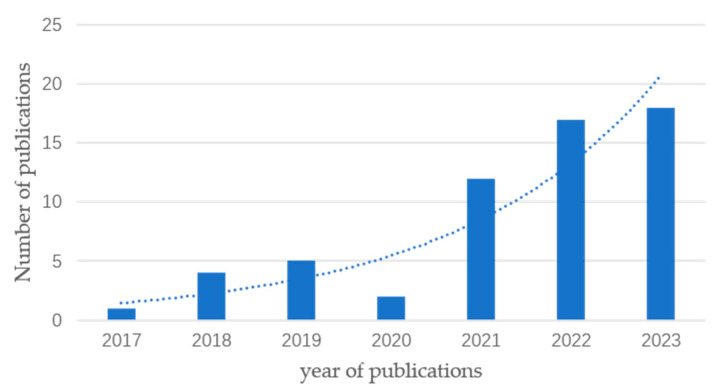
This figure, generated from the annual count of eligible papers, illustrates the growth trend of embodied intelligence in soft robotics over several years.

For each selected paper, we extracted information on embodied intelligence and bionic soft robots. Then, we analyzed and categorized this information to summarize the current state of research on embodied intelligence within the context of soft robotics and to identify trends and gaps in the literature. Based on the number of papers that met the pre-inclusion criteria, a total of 58 papers were selected, dating from 2017 to 2023. Overall, the number of papers indicates that the field of embodied intelligence is still developing and has been increasing annually. This trend is also supported by Figure 2.

## 3. Embodied Intelligence and Its Relationship with Other Intelligences

Artificial intelligence (AI) is a broad concept that includes cognitive intelligence, physical intelligence, perceptual intelligence, computational intelligence, and embodied intelligence, as shown in Table 1.

**Figure 3:**
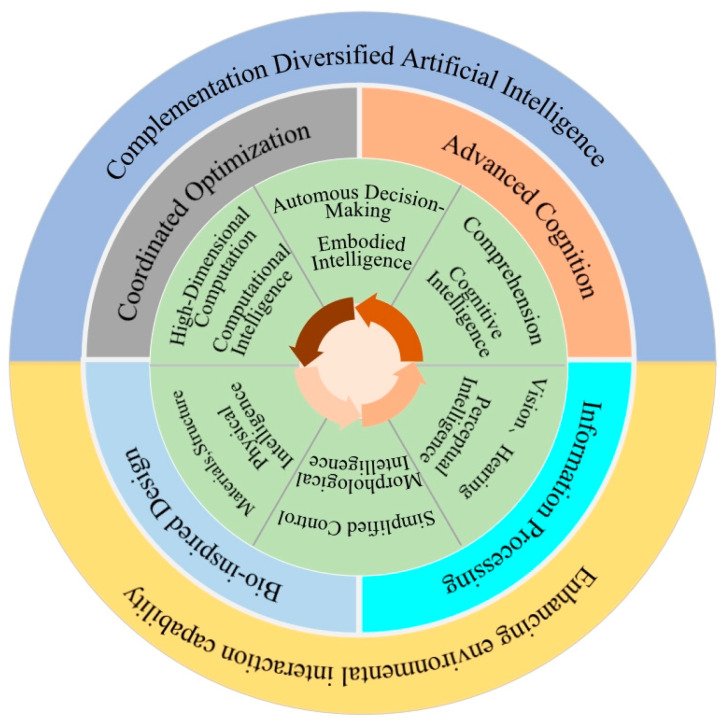
Multilayered and interactive map of diverse intelligence systems.

In the development of artificial intelligence, different types of intelligence complement each other, forming a progressive relationship from the bottom layer to the top layer. Their relationship graph is illustrated in Figure 3. The foundation consists of computational intelligence and physical intelligence, which provide basic algorithmic support and direct interaction capabilities with the physical world. They focus on designing and implementing algorithms for data processing (computational intelligence) and the physical interaction of robots (physical intelligence). The middle layer, composed of perceptual intelligence and cognitive intelligence, further enhances the system’s complexity. Perceptual intelligence allows the system to sense the environment through sensors, while cognitive intelligence emulates advanced human cognitive functions, such as understanding, thinking, and reasoning. The main difference between them is that perceptual intelligence focuses on the acquisition and processing of external information, whereas cognitive intelligence focuses on internal processing and decision making. Morphological intelligence, as a key aspect, focuses on how a robot’s physical form can achieve adaptive interaction with the environment through a morphological computation and a simulation of biological evolution, highlighting the impact of robotic physical characteristics on perception abilities and cognitive processing methods, as well as how to utilize biological evolutionary principles to enhance robots’ adaptability and efficiency. The highest layer, embodied intelligence, integrates all features of the lower and middle layers, combining computation, physical interaction, perception, and cognition within a robot, aiming to achieve complex and dynamic interaction with the environment. The uniqueness of embodied intelligence lies in not only combining the features of all other levels of intelligence but also emphasizing a direct interaction between the robot’s physical form and the environment, thus achieving a more comprehensive and efficient intelligent performance. Compared to general intelligence, features of embodied intelligence include proactivity, enabling intelligent systems to become active participants rather than passive information-processing tools; real-time responsiveness, allowing for immediate reactions to new information or environments; contextuality, akin to how humans adjust their behavior in real-time interaction with their surroundings, and embodied intelligence should profoundly understand its context through real-time learning and feedback and adjust its behavior accordingly; and biomimicry, dynamically adjusting behavior and structure based on environmental changes and interactions to develop higher levels of function and structure, thereby enhancing the system’s robustness and adaptability.

## 4. Research Progress on Embodied Intelligence in the Context of Soft Robotics

This section discusses the progress of research on embodied intelligence in soft robotics. Compared to general artificial intelligence, embodied intelligence is required to cope with complex environments and to coexist with the real world in a way closer to human cognition. This makes it exhibit more biomimetic features. Through millions of years of adaptations and co-evolution, organisms on Earth have developed nervous and musculoskeletal systems to achieve efficient task performance. Mengaldo [18] believes that the softness and compliance of organisms are key to their embodied intelligence. Similarly, Leschi C [19] also believes that embodied intelligence relies on the physical body’s flexibility, generating sensations and behaviors through an interaction with the environment. This interaction adapts to external changes through the soft body while integrating with the actions produced by actuators. This indicates that soft robots contribute to embodied intelligence by enhancing adaptability and perception through their unique softness and compliance. At the level of embodied physical intelligence, Sitti M [20] points out that flexible systems can respond to environmental stimuli. Through this response, they can perceive their own movements and states, such as detecting movements and positions of various body parts, and then they can plan and execute subsequent actions based on these perceptions. This means that embodied intelligence can not only perceive the environment through flexible systems but can also autonomously act based on perception.

Not only are there theoretical hypotheses and reflections, but there have also been various advancements in this research field, including embodied morphological computation; embodied artificial evolution; and perception, control, and decision making in soft robotics. These studies aim to further enhance the adaptability, flexibility, and intelligence of soft robots, making them better suited to complex and evolving environments while also contributing to the refinement of the theoretical framework of embodied intelligence. Here, within the context of soft robotics, we mainly discuss the following issues regarding embodied intelligence:

- How to design computable body morphology: carry out research on how to achieve intended computational functionalities through designing body structure and materials, including optimizing perception, decision making, and behavior generation through morphological design.
- The co-evolution between body and control systems: carry out research on how to co-evolve the body (morphology) and brain (control system) of robots and how these interact to influence the overall performance and adaptability of the robot jointly.
- Exploring how soft robots utilize their flexible bodies and materials to perceive changes in the external environment and how to use this perceptual information for real-time decision making and control, driving the development of soft robotics towards higher intelligence, autonomy, and practicality.

### 4.1. Bionic Soft Robots

Soft robots are considered ideal subjects for the study of embodied intelligence because they exhibit a range of unique characteristics and advantages that are closely related to embodied intelligence: embodied intelligence emphasizes that agents interact with the environment through their physical form, and the soft and flexible structure of soft robots enables them to move and manipulate effectively in complex and irregular environments, mimicking the natural movement and adaptability of living organisms. An agent is defined as a system or entity that interacts directly with its surrounding environment through its physical embodiment to perform tasks and achieve objectives. For self-evolving repair [21], soft robots might be the key to achieving this advanced skill, which can significantly enhance the durability and adaptability of robots in terms of embodied intelligence. For mimicking soft biological sensing mechanisms [22], organisms perceive the external environment through organs like skin and tentacles, integrating pressure sensors and temperature sensors to achieve multimodal sensing for a more comprehensive perception and response to environmental changes. Control of highly integrated systems and execution of high-dimensional continuous action tasks: The control issue of soft robots reflects a key challenge of embodied intelligence, which is how to effectively manipulate a complex physical system in a continuous, dynamically changing environment. This involves a deep understanding of the dynamics of both the physical world and the robot itself.

**Figure 4:**
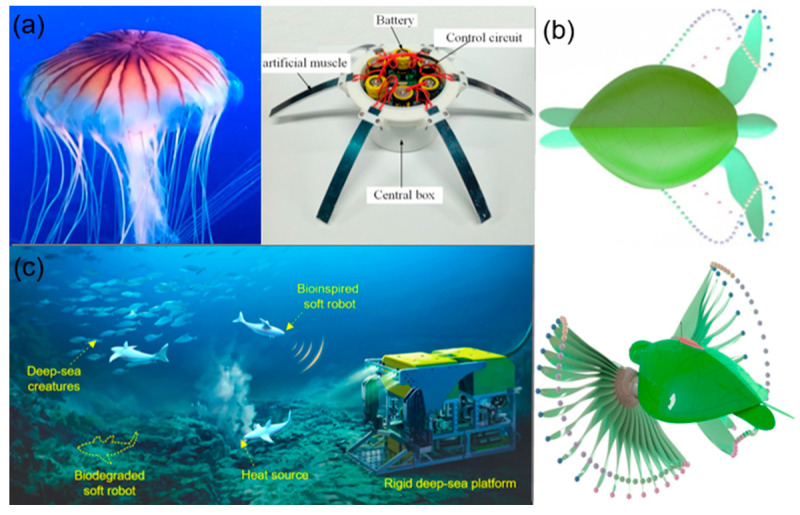
(**a**) Aurelia-inspired robot based on SMA artificial muscle [24]. (**b**) Sea turtle-inspired swimming robot [25]. (**c**) Bionic soft robotic fish investigates deep-sea environments [26]; reproduced with permission from ref. [26], copyright 2023, Springer Nature.

Soft robotics is a multidisciplinary field encompassing branches such as mechanical engineering, biology, electronics, and more, specialized in creating and designing robots. The drawbacks of traditional robots, such as rigidity, complexity, and poor flexibility, have prompted researchers to draw inspiration from nature, delve into biomimetic mechanisms, and develop various types of soft robots [23]. From a biomimetic perspective, the design and implementation of soft robots are inspired by the structures and functions of biological organisms found in nature [24,25,26,27,28,29]. Researchers have designed various types of soft robots, as shown in Figure 4. Many natural organisms, such as octopuses and jellyfish, exhibit remarkable flexibility and adaptability. Soft robots mimic these organisms, enabling effective movement and operation in complex and crowded environments, which are characteristics that embody the essence of embodied intelligence. Moreover, their soft body structures enable energy storage and conversion for efficient movement and long-term task execution [30]. Microelectronic morphogenesis technology and bio-inspired dynamic morphing technology have provided soft robots with the ability to self-organize and self-repair, enhancing their capability to adapt to complex environments [31,32]. By integrating novel sensors and actuators, soft robots have shown significant application potential in the medical and biomedical fields, especially in the precise delivery of cellular cargo, paving the way for new research directions [33]. These advancements not only drive the development of soft robotics technology but also offer important perspectives and tools for a deeper understanding and realization of embodied intelligence.

### 4.2. Embodied Morphological Computing

By employing soft and intelligent materials, soft robotics extends traditional robotic techniques, enabling robots to work with humans and handle delicate objects. However, this potential comes at a cost. Soft robots often exhibit complex dynamics that are challenging to model, making them difficult to control. Interestingly, natural soft-bodied organisms, such as octopuses, have evolved over millions of years to develop efficient methods for utilizing their complex morphological features for perception, control, and computation. This natural strategy has sparked research in morphological computation, a field that explores how to translate this capability observed in biological organisms into robotics technology.

**Figure 5:**
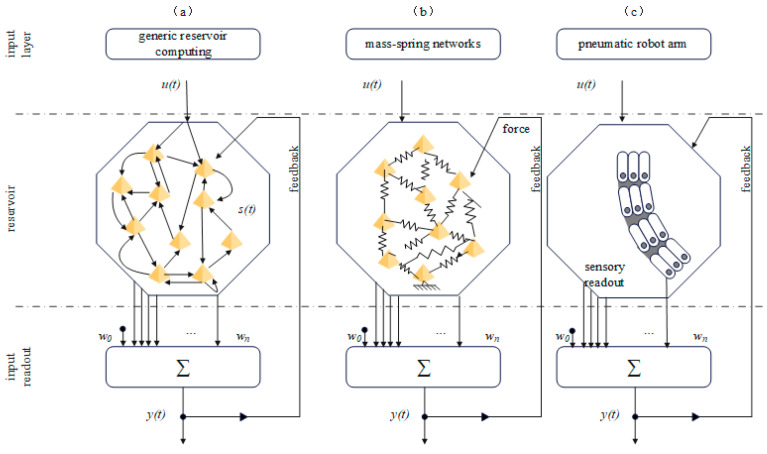
(**a**) General reservoir computing, also known as an echo state network, is considered as an extension framework of neural networks. It shares the same three-layer structure. (**b**) Mass-spring networks are a biomimetic computing model. (**c**) Sampled modular pneumatic soft arm as an energy storage reservoir.

In artificial intelligence and machine learning, reservoir computing (RC) is gradually becoming a topic of great interest. As a unique computing framework, it demonstrates tremendous potential in handling complex time-series data. It is a neural network-based computing approach, with its core being a fixed and randomly generated large-scale neural network known as the “reservoir” [34]. Unlike traditional neural networks, this network does not require comprehensive training but rather maintains its initial random connectivity state, as depicted in Figure 5a. This framework is considered as an extension of neural networks and comprises three main components: The input layer, which can be composed of one or multiple nodes and belongs to the feed-forward neural network category. The middle layer, consisting of multiple nodes and belonging to the recurrent neural network category. The output layer, which is a weighted summer. The reservoir’s function is to transform input data into higher-dimensional dynamic representations, thereby enhancing the data’s nonlinear characteristics and complexity. The only part that needs to be trained is the output section of the network, which significantly simplifies the training process and reduces the demand for computational resources.

Research by Helmut Hauser and colleagues demonstrates how introducing feedback mechanisms into the design of flexible robots enables the autonomous generation of adaptive motion patterns without relying on complex control algorithms [35]. The feasibility of this theory has been validated through computer simulations of mass-spring networks, as depicted in Figure 5b, which simulate the mass and connectivity relationships of objects to form complex network structures. In this simulation, particles represent parts of objects, and springs represent the connections between objects or the constraints between components. By adjusting the parameters of particles and springs, different shapes, structures, and dynamic behaviors of objects can be simulated. This work expands the application of morphological computation in robotics and provides a new perspective for understanding the control principles of biological organisms. Nakajima introduces an innovative concept by using soft structures mimicking octopus tentacles as reservoir computing devices, enabling complex nonlinear behavior simulation without external controllers [36]. This approach utilizes the physical dynamics of soft tentacles to simulate information processing through intrinsic physical responses, achieving the capability of closed-loop control.

Further research indicates that morphological computation can extend to environmental perception and localization. Eder M’s study demonstrates the potential of soft robotics in simulating and learning complex dynamic control processes through pneumatic-driven modular manipulator arms [37], as shown in Figure 5c. This approach, which utilizes the structural and dynamic properties of soft robots for learning and control, not only enhances the flexibility and accuracy of robot operations but also demonstrates the possibility of achieving computation and control through physical morphology. Research by Judd et al. utilizes the body dynamics of soft robots to predict the position of objects in water, showcasing the potential of morphological computation in perception and interaction [38]. This body-dynamics-based perception method not only reduces dependence on traditional sensors but also enhances the adaptability and responsiveness of robots to the environment.

These research findings collectively point to a consensus: the integration of soft robotics and morphological computation provides a new direction for developing highly adaptive and intelligent robotic systems. This direction not only challenges traditional paradigms of robot design and control but also offers valuable insights for understanding biological intelligence mechanisms and developing a new generation of bio-inspired robots. The morphological computation methods and design concepts discussed in this section are innovative, but they are difficult to form relatively systematic design schemes and lack the ability to quantitatively evaluate the role of morphological computation. If they can be integrated with other disciplines to form better design solutions and applied to soft robotics or medical fields, this will be a very promising research direction.

### 4.3. Embodied Artificial Evolution

**Figure 6:**
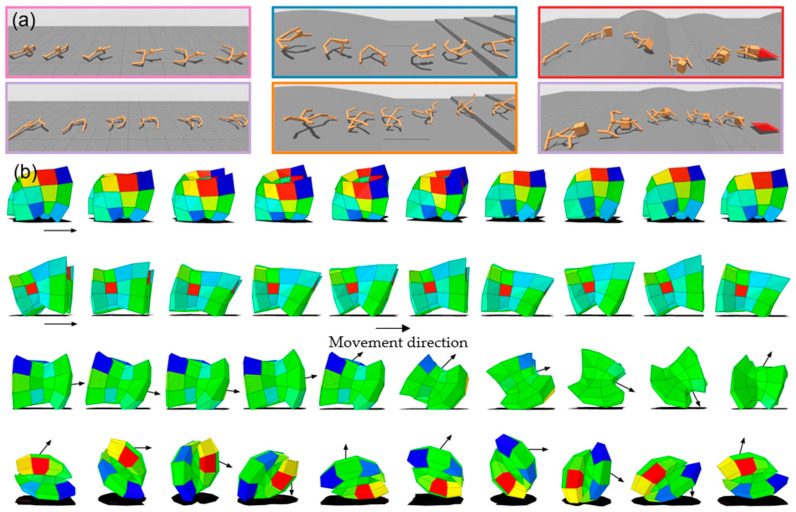
(**a**) Evolution of intelligent agents in different terrains. Reproduced with permission from ref. [42], copyright 2021, Springer Nature. (**b**) Each row depicts a different evolved robot moving from left to right. Voxels in the figure are colored based on the amount of subsequent morphological development remaining in that cell: blue indicates shrinking voxels, red indicates growing voxels, and green indicates minimal change. The first row features an evolved soft quadrupedal robot trotting with a two-beat gait synchronizing diagonal pairs of legs. The second row shows an adult robot galloping at full speed (fully airborne mid-gait). The third row depicts a juvenile robot galloping at full speed, evolving into an adult form capable of rolling. The fourth row showcases a rolling robot. Reproduced with permission from ref. [44], copyright 2018, Springer Nature.

From the perspective of biological evolution, all organisms interact with their surrounding environment(s) through their physiological characteristics, achieving lifelong learning and continuous evolution. Evolution is a long-term process based on adaptive selection in the environment [39]. The research of translating natural evolution into artificial evolutionary algorithms has made significant progress. Taking it a step further, extending the artificial evolution process from digital space to physical space is referred to as “embodied artificial evolution” [40]. Feng et al. [41] defined the “genes” of machines as “learning genes” and proposed genetic reinforcement learning (GRL), simulating the evolution of organisms in physical space. They utilize learning genes to train and evolve intelligent agents, which demonstrate superior performance in various tasks and scenarios, showing higher efficiency and effectiveness. In 2021, the team led by Fei-Fei Li [42] developed an embodied intelligence computing framework (DERL) by combining evolutionary algorithms with deep reinforcement learning. The aim was to evolve diverse agent morphologies capable of learning complex locomotion and manipulation tasks in different environments (Figure 6a). This study also demonstrated the Baldwin effect for the first time, which suggests that behaviors learned by individuals in the early stages of evolution may gradually become instinctive and even be passed on to offspring. Similarly, Saito, Takumi et al. [43] confirmed the influence of body movements on the evolution of body shape using two-dimensional soft robots. They found that body morphology significantly affects the learning of movement, which is consistent with the theory of embodied intelligence. Research on embodied intelligence focuses on the brain controlling the interaction between the body and the environment and involves collaboration between the brain and the body. Luo J [44] studied the integration of learning into morphological evolution in robot systems. They utilized central pattern generators (CPGs) to control modular robots and employed evolutionary algorithms to search for robots with better fitness (as shown in Figure 6b). This approach enabled the simultaneous evolution of the robot’s morphology (body) and controller (brain), incorporating a learning phase to optimize the inherited brain for better control of the robot’s body. In this process, the changes in the body affect the learning and adaptation of the brain, thereby indirectly influencing the evolution of the brain. In another related study, Pathak D [45] used graph dynamic networks to encode modular robots, defining a morphological search as a reinforcement learning Markov process. Trained agents can assemble their limbs and obtain morphologies adapted to specific environments (such as underwater environments or stair-like terrain) through a morphological search.

Through evolutionary algorithms and reinforcement learning, researchers can simulate and explore how different body shapes affect the learning and behavior of intelligent agents and how these body shapes further adapt to the environment, deepening our understanding of the theory of embodied intelligence. The core idea of embodied intelligence is that intelligence arises from the dynamic interaction between organisms and their environment, meaning that intelligence is not confined solely to the brain or control systems but is the result of the entire body’s interaction with the environment. In a natural evolution process, organisms’ morphology and behavioral control co-evolve and interact. By combining evolutionary algorithms and reinforcement learning, it is possible to simultaneously optimize the design of a robot’s morphology and control strategies, enabling them to work together more effectively and improve the overall performance and adaptability of the robot.

**Figure 7:**
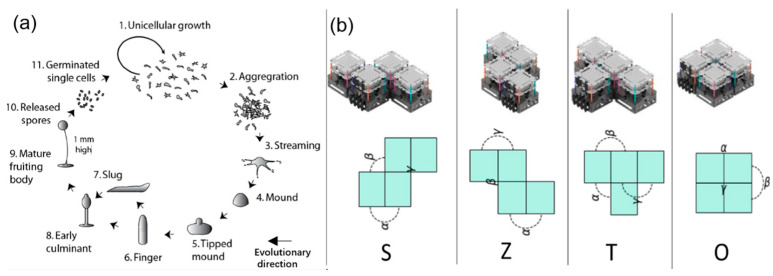
(**a**) *Dictyostelium discoideum*, commonly referred to as slime mold, capable of a transition from a collection of unicellular amoebae into a multicellular organism [46]; (**b**) modular reconfigurable robots [47]. Reproduced with permission from ref. [47], copyright 2022, Springer Nature.

In the biological realm, the evolution of self-organizing multicellular organisms represents a remarkable transition from single-celled to multicellular life. Multicellular organisms provide inspiration for the adaptation and evolution principles in robotics technology. Especially known as multi-robot organisms, these are defined as large-scale collectives of robots that can dock with each other within a single “artificial life form” and symbiotically share energy and computational resources. Doing so is advantageous, as these robots can dynamically aggregate and self-assemble into one or multiple symbiotic organisms, enabling them to interact collaboratively with the physical world through various sensors and actuators. Artificial multi-robot organisms represent research into adaptive, reconfigurable, and collective robotic systems. Inspired by biology, examples of such systems can be found in nature, with one of the most famous being Dictyostelium—social amoebae, which also known as cellular slime molds [46]; these soil-living unicellular amoebae feed on bacteria. When food resources are depleted, the amoebae produce and release signaling molecules called cAMPs. This chemotactic mechanism creates a gradient field towards an aggregation point, causing up to 100,000 cells to aggregate into a multicellular organism, forming a fruiting body, as shown in Figure 7a. In this process, the amoebae undergo various developmental stages such as cell differentiation, morphogenesis, growth, self-protection, and sexual and asexual reproduction. The principles underlying self-motion, aggregation, and emergent macroscopic functionality can also be demonstrated through artificial systems, particularly swarm robotics. Like amoebae, swarm robots can emit aggregation signals and assemble into artificial organisms [47], as shown in Figure 7b.

According to the concept of developmental plasticity, organisms can self-assemble and self-disassemble. Specifically, this implies that artificial organisms have two main distinct states—collective mode and organism mode—and undergo several stages including collective behavior, self-assembly, homeostasis regulation, and macroscopic coordination. In the future, robots are sure to cooperate with other robots just like these intelligent organisms.

### 4.4. Perception, Control, and Decision Making

#### 4.4.1. Multimodal Perception

Cewu Lu mentioned at the Valse2022 conference that embodied intelligence is not a computer in the traditional sense, it is a multimodal intelligent system. Of course, achieving better physical interactions requires more comprehensive perception technologies. Currently, flexible multimodal sensing technology is a key innovation closely linked to the concept of embodied intelligence. This technology is dedicated to enhancing the perceptual capabilities of robots in their interactions with the environment. Liu, W [48] and colleagues, based on the principles of triboelectric nanogenerators and the piezoresistive effect of liquid metals, have proposed a flexible bimodal smart sensor (FBSS) capable of real-time sensing of both non-contact and contact signals. The FBSS can differentiate between tactile (physical contact) and non-tactile (proximity) interactions. Researchers have demonstrated the capability to remotely instruct a soft robotic arm to complete operational tasks without touching it and without wearing any devices, achieving this through air-gesture teaching. Building on this, Ham, J also developed a sensor (BSFS) that operates in both non-contact and tactile modes for a soft robotic arm, based on triboelectric nanogenerators and the giant magneto strictive effect [49]. This dual-mode sensor is used to detect the physical characteristics of target objects, such as shape, material, and surface roughness. Using machine learning methods, specifically convolutional neural networks, the system achieves up to 97% accuracy in identifying the attributes of objects. Finally, the characteristics of the objects are succinctly described through natural language information transmitted via screens and speakers. Furthermore, the field of soft robotics has explored biological sensing mechanisms to better understand the interaction with external physical information during morphological changes. This includes flexible pressure sensors [50], flexible temperature sensors, and flexible tactile sensors.

#### 4.4.2. Control Strategies

**Figure 8:**
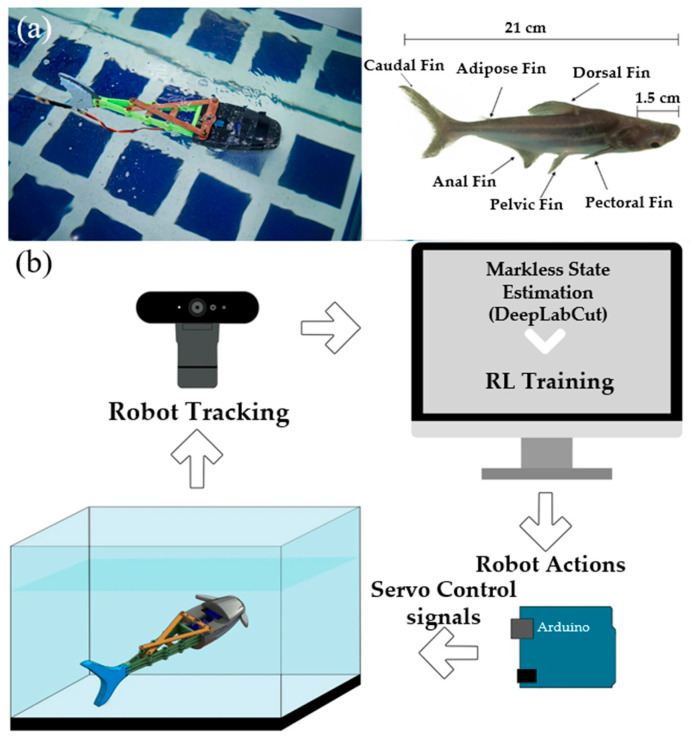
(**a**) Soft-bodied biomimetic robotic fish. (**b**) A schematic diagram of reinforcement learning-based control. The environment frames captured by the camera at a speed of 60 fps are sent to DeepLabCut for pose estimation to obtain the precise position and posture of the robotic fish. This precise position and posture information is then used as input for the reinforcement learning algorithm to train the soft-bodied biomimetic robotic fish to reach the target [54]; reproduced with permission from ref. [54], copyright 2023, Springer Nature.

Embodied intelligence robots emphasize the need to design more powerful and flexible controllers to better facilitate the autonomous operation and adaptability of soft robots. The dynamic modeling and solving for soft robots are typically complex computational tasks, particularly in scenarios with a high degree of freedom (DoF) and complex morphologies. This complexity arises because soft robots, unlike their rigid counterparts, can deform in infinite ways, which requires sophisticated models to predict their behavior accurately and control strategies that can adapt to a wide range of conditions and tasks. Model-free control methods, such as deep reinforcement learning, enable learning through real-time interactions with the environment. These methods can optimize the morphology of soft robots, making them better suited to specific task requirements, such as changing shape to grasp or envelop objects. Currently, many standard control approaches are open-loop control. Centurelli A et al. proposed a neural network-based closed-loop controller trained using a deep reinforcement learning algorithm called Trust Region Policy Optimization (TRPO) [51]. They conducted experiments on a soft robotic arm, preliminarily confirming the feasibility of using deep reinforcement learning for controlling soft robotic arms. After completing the first step of closed-loop control, Agabiti, Camilla A et al. [52] devised a grasping strategy based on identifying contact points on objects to force the arm to bend and induce wrapping around the object. They verified in a simulated environment that a reinforcement learning strategy fused with an finite element simulation can induce deformation of the soft robotic arm to wrap around objects and perform grasping. Soft robots have more passive degree of freedom than rigid robots, posing challenges for controller design. Ryota Morimoto [53] proposed an integrated lightweight model-free reinforcement learning network called ELFNet. ELFNet utilizes N Q-networks and target networks, as well as M policy networks, which help to mitigate overestimation and underestimation in reinforcement learning, enabling a more accurate estimation of action values. Experiments involving continuous manipulation of a robot arm’s end effector to find a target position confirmed that this model-free algorithm is more suitable for controlling soft robotic manipulators than other reinforcement learning algorithms. For more complex control tasks, Youssef, S.M. utilized a fin-ray tail structure driven by servo motors to mimic the undulating swimming motion of fish [54]. They employed reinforcement learning as a model-free control strategy, enabling the robotic fish to swim effectively and reach specified targets, demonstrating the robot’s learning ability to perform required tasks in an aquatic environment, as shown in Figure 8. To investigate morphological learning control, many researchers have proposed simulation environments tailored for reinforcement learning of soft robots based on existing robotics learning environments. Examples include SofaGym [55], Elastica [56], SoMoGym [57], Evolution Gym [58], SoftCon [59], and others.

#### 4.4.3. Autonomous Decision Making

Autonomy is crucial for robots in applications such as search and rescue, surveillance, and patrol missions. It is also one of the goals in the future of embodied intelligence, where robots are capable of making autonomous decisions akin to human cognition. To achieve this goal, researchers are actively exploring integrating artificial neural networks with soft robotics. In autonomous decision making and navigation, S. Bai et al. achieved autonomous exploration and mapping of mobile robots through deep neural networks [60]. In autonomous operation, Jitosho R implemented real-time agile operations autonomously on pneumatic actuator soft arms using deep reinforcement learning algorithms [61]. In more complex dynamic scenarios, Zhang R proposed a behavior intention recognition network based on a self-attention mechanism. The self-attention mechanism weights the input features to highlight important information, thereby improving the accuracy and efficiency of the recognition process. Furthermore, to enable robots to better adapt to changes in human–robot interaction environments, reinforcement learning algorithms were introduced into the robot’s control system. By continuously learning from environmental feedback, the robot can autonomously optimize its decision-making process, achieving efficient adaptive control in complex dynamic scenarios [62].

To enable robots to make rapid and accurate decisions in continuously changing environments presents a significant challenge. A centralized evaluation of decisions becomes crucial yet complex in this process. Currently, several approaches address this challenge. Firstly, through reinforcement learning and simulation training, robots can be trained in simulated environments, allowing them to explore various strategies in a safe virtual setting and optimize decision-making processes through reinforcement learning algorithms. For instance, the soft actor-critic algorithm employs gradient-based methods to update its parameters, enabling robots to make optimal decisions in diverse scenarios [63]. Secondly, utilizing decision trees and path planning algorithms assists robots in evaluating different decision paths and selecting the most optimal route to reach objectives [64]. Decision trees form tree structures by analyzing problems and abstractly describing datasets through entropy reduction and inferring strategies. Furthermore, modularizing complex decision-making processes into manageable modules and ensuring their effective integration and collaboration enhances overall system efficiency and effectiveness. Lastly, multi-sensor data fusion integrates inputs from various sensors such as cameras, radars, and laser rangefinders, providing robots with a comprehensive understanding of their environment for improved decision making. Fusion technologies like Kalman filters and particle filters play pivotal roles in this process. Thus, integrating these methods and strategies is essential to enhance robot autonomy and decision-making capabilities in dynamic environments [65].

## 5. Summary and Future Challenges

We explore the field of embodied intelligence in soft robotics, analyzing typical cases and summarizing research progress across different branches. Significant progress was made in three main areas: embodied morphological computation, embodied artificial evolution, and soft robotics in perception, control, and decision making. It is important to emphasize that exploring how to achieve more natural and effective robotic behavior through physical forms and intelligent interaction represents the state-of-the-art of embodied intelligence research. From the perspectives of morphological computations, interactions with the environment, and the integration of perception, control, and decision making, soft robots offer an excellent platform for research in embodied intelligence.

However, there are still many challenges in these areas. Firstly, a morphological computation must consider not only physical and mechanical properties but also their alignment with task requirements. Secondly, it must consider how to maximize computational capacity, sensory capabilities, and energy efficiency through morphological design. Finally, deploying these capabilities in a completely soft robot capable of independently completing tasks requires interdisciplinary collaboration and innovative design methods. The co-evolution of soft robotic morphologies (bodies) and controllers (brains) presents an even more complex challenge. This necessitates designing physical forms adaptable to diverse environments and tasks and developing algorithms and control strategies that can effectively manage these forms. Progress in experimental validation and simulation technology is also needed. Emphasizing the direct interaction with the environment within the framework of embodied intelligence, soft robots require deep integration of perception, control, and decision making, making full use of reinforcement learning tools and their capabilities for model-free control and interactions with the environment. Addressing the multifaceted challenges of soft robotics and embodied intelligence also demands close cooperation among fields such as robotics, material science, biology, computer science, and ethics.

## Figures and Tables

**Table 1:** The definition of embodied intelligence compared to other forms of intelligence.

Noun	Definition	Emphasis
Computational Intelligence	A method imitating natural intelligence, including neural networks, evolutionary algorithms, fuzzy systems, and machine learning [11,12,13].	Solving complex computational problems.
Physical Intelligence	Encode sensing, actuation, control, logic, and computing intelligence into the robot’s body [14].	Reduce costs. Response speed. Enhance robustness.
Perceptual Intelligence	Perceptual intelligence allows machines to sense and interpret the environment, covering senses like sight, hearing, and touch [15].	Accurate information acquisition.
Cognitive Intelligence	The ability of machines to simulate or mimic human cognitive behaviors, including understanding, thinking, and reasoning [16,17].	Enabling machines to understand and utilize knowledge.
Morphological Intelligence	An intelligent robot’s shape affects how it interacts with its surroundings and its smart actions.	Used for simplifying control and data processing.
Embodied Intelligence	Emphasizing the interaction with the environment, integrating complex processes such as perception, learning, decision making, and action, surpassing mere physical movements.	Intelligent systems tightly integrate with their physical environment.

## Data Availability

Data are available in this manuscript.
